# Marketing health by geographic location: improving awareness of the New Mexico Double Up Food Bucks program

**DOI:** 10.1186/s40795-026-01261-z

**Published:** 2026-02-18

**Authors:** Luotao Lin, Bryan Crawford-Garrett, Sonja Baca, Fiorella Viccina, Kathryn E. Coakley

**Affiliations:** 1 Nutrition and Dietetic Program, Department of Individual, Family, and Community Education, the University of New Mexico, Albuquerque, NM 87131 USA; 2 New Mexico Farmers’ Marketing Association, Santa Fe, NM 87505 USA; 3 College of Population Health, the University of New Mexico Health Sciences Center, Albuquerque, NM 87131 USA

**Keywords:** Double up food bucks, Marketing strategies, Mass media, Food incentive program, Advertising

## Abstract

**Background:**

An estimated 26% of eligible New Mexicans participated in New Mexico Double Up Food Bucks (DUFB), a nutrition incentive initiative that provides SNAP participants an additional dollar for every dollar spent on eligible local foods. This study evaluated awareness and perceived effectiveness of DUFB program marketing strategies, tactics, and tools (tools) among adult Supplemental Nutrition Assistance Program (SNAP) participants in New Mexico and compared awareness and perceived effectiveness by geographic location.

**Methods:**

An online cross-sectional self-administered survey was used to evaluate the effectiveness of 10 DUFB marketing tools such as radio advertisements, social media, handouts and posters, and text messages. Survey respondents’ sociodemographic characteristics, perspectives of tool effectiveness, and barriers to DUFB participation were collected, calculated, and compared by geographic location (metro vs. nonmetro) using SAS.

**Results:**

A total of 1061 New Mexico SNAP participants provided valid responses to the survey; of those, 65% were aware of DUFB and 40% had participated in DUFB. The majority (> 72%) agreed or strongly agreed each of the 10 marketing tools would encourage them to participate in DUFB, with significant differences observed between metro and nonmetro participants in their perceptions of the effectiveness of posters (70% vs. 77%), token signage (74% vs. 81%), social media videos and posts (78% vs. 86%), bus ads (78% vs. 84%), and the DUFB website (82% vs. 88%). The most common barrier reported by SNAP participants who had never used DUFB was that they did not know about DUFB (57%).

**Conclusion:**

SNAP participants in New Mexico perceive current DUFB marketing tools as effective; however, marketing strategies should be tailored by geographic location to ensure significantly more SNAP participants know about and can access the program.

**Supplementary Information:**

The online version contains supplementary material available at 10.1186/s40795-026-01261-z.

## Background

The New Mexico Double Up Food Bucks (DUFB) program is a healthy food incentive program that is available to all Supplemental Nutrition Assistance Program (SNAP) participants in New Mexico [1]. Managed by the New Mexico Farmers’ Marketing Association (NMFMA) in partnership with the New Mexico Department of Agriculture and the New Mexico Human Services Department, DUFB doubles the value of SNAP benefits at farmers’ markets, farm stands, grocery stores, and other participating retailers to purchase locally produced vegetables, fruits, legumes, fresh cut herbs, and plants that produce food [1]. For every dollar spent on eligible items, participants receive an equal amount in DUFB as tokens at farmers’ markets, or are automatically credited by the cashier at grocery stores and farm stands, effectively doubling their purchasing power [1]. The DUFB program is automatically available to all New Mexico SNAP participants, requiring no separate sign-up process. DUFB started in New Mexico in 2010 with just 17 farmers’ markets and has grown to over 90 locations statewide [1].

Though more than 25 states participate in DUFB nationally, only six states have reported DUFB outcomes (dietary intake, barriers, facilitators, food and nutrition security status, economic impacts), including New Mexico [2–13]. Findings consistently demonstrate that DUFB not only enhances participants’ fruit and vegetable intake and food security, but also benefits food producers by increasing sales and revenue [2–13]. DUFB therefore addresses a major food access barrier that populations with low-income face by lowering the cost of fruits, vegetables, and other nutrient-dense foods [14]. Improving access to nutrient-dense foods among populations with low-income is important because SNAP participants have lower diet quality, less access to fruits and vegetables, and are disproportionately targeted by unhealthy food marketing [15–17]. 

According to state administrative data, approximately 131,444 New Mexicans participated in DUFB in 2022 [18], representing just 26% of SNAP participants in New Mexico [19]. Therefore, more than 374,000 New Mexico SNAP participants did not participate in the DUFB program despite being eligible. In addition, New Mexico’s Income Eligibility Guidelines for SNAP and Financial Assistance increased from 165% to 200% of the federal poverty level in October 2024 [20, 21], expanding SNAP and DUFB eligibility to a much larger portion of the population.

One potential reason for low DUFB participation is limited awareness of the program among SNAP participants. A white paper published in 2016, for example, showed that the New Mexico DUFB program has low awareness among SNAP participants [22]. Evaluations in other states indicate insufficient marketing reach could be the primary reason that SNAP participants are unaware of the DUFB program [7, 8] A national evaluation also emphasized the importance of marketing strategies, tactics, and tools in promoting the use of food incentive programs such as DUFB and a recent systematic review found marketing assistance could enable SNAP participation [23, 24].

Certain marketing tactics and tools could be particularly useful to increase awareness of DUFB including billboards, grocery store signage, bus stop signage, social media, and word-of- mouth [25, 26] Social marketing strategies to engage SNAP participants have also proven effective such as social media messages through local community organizations, schools, and food banks [14]. Previous studies show that social marketing campaigns reach a large proportion of target populations and increase awareness and engagement with food assistance programs and nutrition information due to the use of trusted community channels, high exposure to the potential participants, and incorporating engaging formats like videos and infographics [27–29]. There is, however, a lack of information on effectiveness of DUFB marketing tactics and tools nationwide, as DUFB studies to date have focused on program impacts on SNAP participants and participating vendors, as noted above, while marketing studies have looked at outreach and awareness for SNAP more broadly. Differences in effectiveness of DUFB marketing tactics and tools by geographic location are also unreported. Metropolitan (metro) areas typically have greater access to digital platforms and more diverse media channels, while non-metropolitan (nonmetro) areas may rely more on word-of-mouth, local organizations, and print media. Investigating potential differences in the effectiveness of marketing strategies between metro and nonmetro areas could inform tailored marketing strategies to maximize awareness of and participation in DUFB. Therefore, the objective of this study was to assess awareness and perceived effectiveness of current DUFB marketing strategies, tactics, and tools among SNAP participants and to compare awareness and effectiveness by geographic location (metro vs. nonmetro) in New Mexico. A secondary aim was to identify barriers SNAP participants face in using DUFB.

## Methods

### Study design

This cross-sectional mixed-methods study was a partnership between a team of researchers at the University of New Mexico in the Southwest region of the United States (U.S.) and the NMFMA. This study design allows for quantitative assessment of program reach and effectiveness of marketing tools within a single time frame [30]. Data were collected through a statewide survey of SNAP participants in New Mexico in September and October 2024. Interviews were also conducted with 10 current SNAP participants; qualitative findings will be reported separately. The study protocol was approved by the Institutional Review Board at the University of New Mexico (IRB-2405129547).

### Participant eligibility and recruitment

Adult SNAP participants in New Mexico were eligible to participate in the study (Table 1). Survey participants were recruited between September 3 and October 20, 2024, through a variety of methods listed in Table 1. All recruitment materials included a direct link and QR code to the study survey, described below. All participants were asked to review the consent form and provided consent to participate by starting the survey. Participants had the option to enter a random drawing to win one of five $50 Amazon gift cards after completing the survey.

**Table 1:** Participant recruitment methods and eligibility criteria for this study

Participant Recruitment Methods	Eligibility Criteria
• GoodFoodNM, the NMFMA’s healthy eating text message program • Social media (the New Mexico Health Care Authority and the NMFMA Facebook pages) • Flyers posted at eight farmers’ markets across the state	• At least 18 years of age • Currently residing in New Mexico • Current SNAP participant • Fluent in English or Spanish

### Survey development

The study survey (Supplementary file) was developed iteratively, with extensive feedback from the NMFMA and DUFB program leadership due to their first-hand experience administering DUFB for more than a decade, including management of program implementation and annual participant impact and feedback surveys as well as several qualitative studies with SNAP and DUFB participants over the years. This first-hand experience helped strengthen the survey’s content validity. Survey questions first assessed awareness of and participation in the DUFB program. Next, participants were asked about awareness and perspectives of 10 DUFB marketing tactics and tools that were implemented in New Mexico between 2016 and 2024. The survey included a combination of closed-ended and open-ended questions. These tactics and tools included: radio advertisements, community posters, DUFB token signage displayed at markets, handouts, a local food guide, social media posts and videos, a bus advertisement, a billboard advertisement, the New Mexico DUFB website, and GoodFoodNM text messages. All marketing tactics and tools are currently in active use except for the bus advertisement. Most marketing tactics and tools were available in both English and Spanish. Graphics or links to audio and video samples were provided to survey participants for all tactics and tools evaluated (Table 2). Since bus ads were no longer available and billboards were implemented three days prior to survey distribution, the percent of survey respondents who had seen these tools was not evaluated in the survey.

**Table 2:** New Mexico DUFB marketing tools and tactics evaluated

Marketing tools and tactics	Examples	Targeted areas	Timeline	Target audiences and tool/tactic purposes
DUFB radio ads	English Spanish	*2024 locations*: • KNCE - Taos and surrounding area • KDCE - Española and surrounding area (Spanish) • KISZ - Farmington and all of San Juan County • KABG (Big 98.5) - ABQ., Santa Fe, Rio Arriba, Taos, Los Alamos, Pecos, Las Vegas • KKRG (Mix 105.1) - ABQ, Santa Fe, Crownpoint, Rio Arriba, Peñasco, Taos, Los Alamos, Pecos, Las Vegas, Cuba • KKSS (Kiss 97.3) - ABQ, Mountainair, Santa Fe, Las Vegas. Pecos, Questa, Angel Fire • KLVO (Radio Lobo) - ABQ., Socorro, Grants, Santa Fe, Jemez Springs, Edgewood, Pecos • KABG (102.9 Fuego) - ABQ, Santa Fe, Rio Arriba, Taos, Los Alamos, Pecos, Las Vegas • KYVA, KXXI, KYAT, & KYVA – Gallup (Navajo language) • KVLC (101 Gold) - Las Cruces and Deming • KXPZ (99.5) - Las Cruces and surrounding area • KMVR (104.9) Magic - Las Cruces and surrounding area *Prior to 2024 locations*: **In metro counties**: • Farmington/Aztec •Albuquerque • Las Cruces • Santa Fe **In non-metro counties**: • Espanola (Spanish language) • Gallup (Navajo language)	Since 2015	Targeting general population in select geographic broadcast areas and used for: • Program awareness • Reasons to believe (save money, eat more produce) • Where to shop
DUFB community posters		All counties with participating DUFB outlets in New Mexico (25–27 counties per year), distributed via participating outlets and community partners	Since 2015	Targeting potential DUFB shoppers and used for: - program awareness and basic information; - promoting local food
DUFB token signage		At all DUFB outlets in all counties with participating outlets in New Mexico (25–27 counties per year), distributed via all participating outlets	This version since 2023, previous version since 2015	Targeting SNAP and Double Up shoppers and used for program awareness
DUFB handouts		All counties with participating DUFB outlets in New Mexico (25–27 counties per year), distributed via community partners (e.g., farmers’ markets, libraries, food bank and food pantry, Income Support Division offices, and Cooperative Extension offices)	Since 2015	Targeting SNAP participants, used for: • Program awareness and how it works • Where to shop
Local food guide		All across New Mexico, distributed via community partners (e.g., farmers’ markets, libraries, food bank and food pantry, Income Support Division offices, and Senior centers)	Annually since 2023	Targeting general NM population and used for: • Program awareness • Providing participating outlet directory with locations, operating days/hours, and other helpful information
DUFB social media posts and videos	Video 1 Video 2	All across New Mexico	Videos released 2018 Social media posts started in 2015	Targeting general NM population and used for: • Program awareness • Education about program availability, seasonality, and maximizing benefit • Reasons to believe (save money, eat more produce)
DUFB bus ad		Albuquerque, NM	2016–2022	Targeted Albuqurque population and visitors, used for program awareness
DUFB billboard		Albuquerque, NM	2024-present	Targeting Albuquerque population and visitors, used for program awareness
DUFB NM website	www.doubleupnm.org	All across New Mexico	Since 2015	Targeting SNAP users in NM and used for: • Program awareness • Where to shop • Reasons to believe (save money, eat more produce)
GoodFoodNM text messages		All across New Mexico	Since 2020	Targeting: • English and Spanish-speakers • SNAP & DUFB participants • WIC participants • Families with children • Produce prescription program patients • Farmers’ market customers Used for: • Empowerment/Choice messages • Family Support messages • Control/Practical Steps messages • Nutrition information • Recipe links • Seasonal food info • Tips for shopping, cooking/prep, food storage/preservation

Survey participants who had participated in DUFB (DUFB group) answered one set of survey questions about their experiences, perspectives, and suggestions. Meanwhile, those who were SNAP participants but had never participated in DUFB (SNAP group) answered another set of questions about their understanding and perspectives of the program, suggestions for improving the program’s marketing and communication efforts, and barriers to participating in DUFB. Both groups provided demographic information at the end of the survey including gender, age, race and ethnicity, highest education level, annual household income, number of adults and children in the household, and county of residence that followed U.S. Census Bureau categories [31, 32] Respondents were allowed to select more than one option for race and ethnicity and were categorized as “White, not Hispanic”, “African American, not Hispanic”, “Hispanic, any race”, “Asian, not Hispanic”, “Native American, not Hispanic”, “Multiple races reported, not Hispanic”, and “Race unknown” to align with USDA SNAP participant reporting [33]. In addition, two attention check questions were included in the survey to ensure participants were reading questions and providing legitimate answers.

The final survey included 62 questions for the DUFB group and 58 questions for the SNAP group. Of these, 41 questions were mandatory for the DUFB group and 37 for the SNAP group, with the remaining questions being optional. The survey was translated to Spanish by a native speaker on the research team and back-translated to ensure content accuracy and cultural appropriateness. Participants could choose which language they preferred to complete the survey. This manuscript reports the specific subset of questions related to marketing of DUFB (54 questions for the DUFB group and 52 questions for the SNAP group).

The survey was self-administered online through Qualtrics. After reading the consent form and providing consent by starting the survey, participants completed four questions assessing eligibility based on age (18 years or older), New Mexico residency, and self-reported current SNAP participation status; those who were not eligible to participate did not complete the rest of the survey (*n* = 313, 10%).

### Data cleaning

**Fig. 1:**
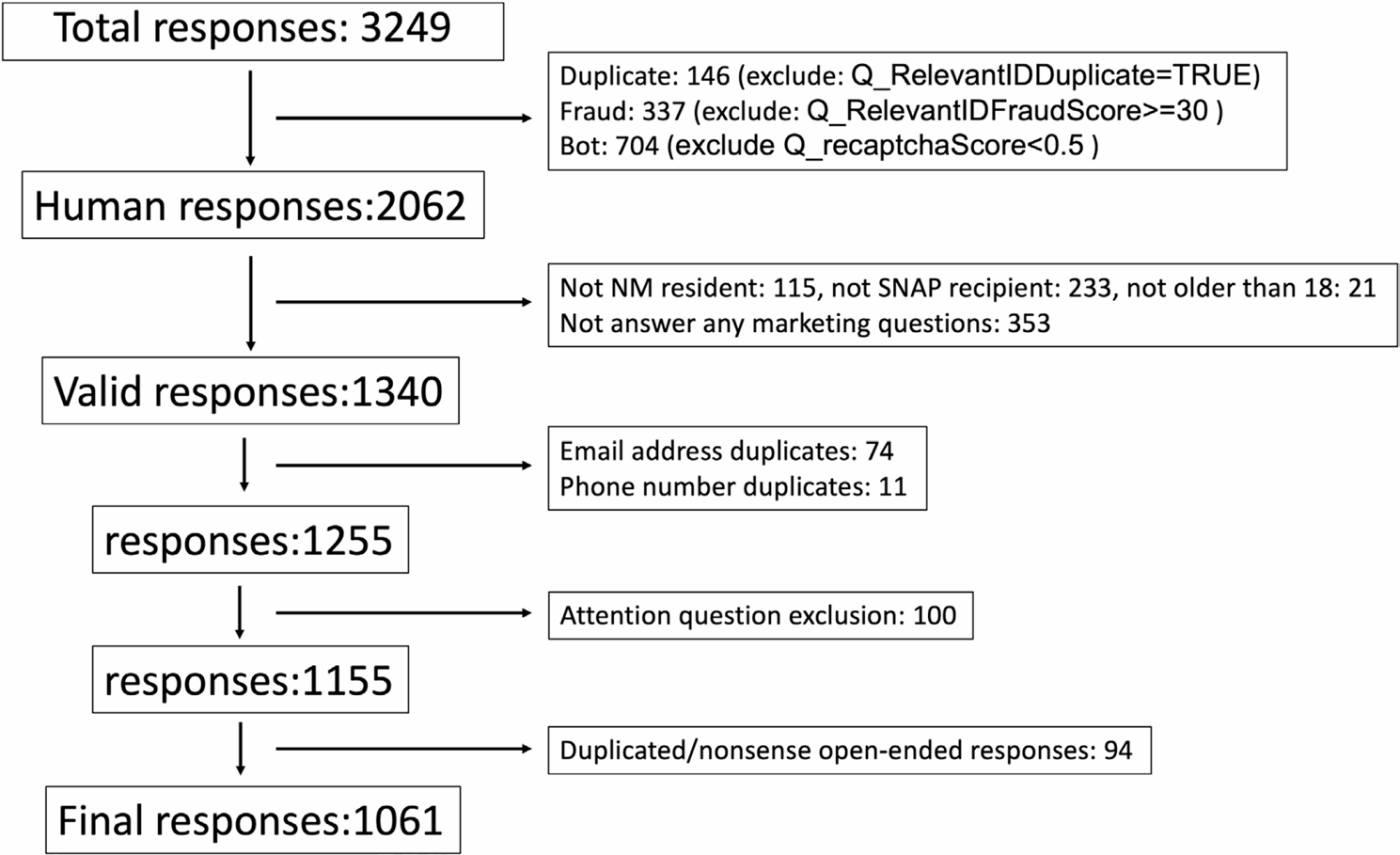
Flow chart of participants in this study

After the survey closed on October 20, 2024, survey data were downloaded to an excel file for data cleaning. A total of 3,249 responses were received. Qualtrics’ bot and fraud detection metrics were first used to exclude suspicious responses, including participants with a reCAPTCHA score < 0.5 (suggesting likely bot response), a duplicate score of > 75 or duplicate ID of “true”, or a fraud score of > 30 (*n* = 1187). Next, participants that did not complete screening questions or that were ineligible were excluded (*n* = 369) followed by those that did not evaluate at least one marketing tactic or tool (*n* = 353). Then, responses with the same email address or phone number provided at the end of the survey were excluded (*n* = 85) and attention check questions were used to exclude another 100 participants. Finally, responses to open-ended questions were reviewed by the research team for duplicate text and/or nonsense responses resulting in the exclusion of an additional 94 participants. After excluding ineligible and suspicious responses (*n* = 2,188, 67.3%), 1061 unique participants (32.7%) were included in the final analytic sample. (See flow chart in Fig. 1).

### Data analysis

Quantitative survey data were analyzed using SAS. Descriptive statistics were calculated and compared for all variables by metro vs. nonmetro residence, including frequency and percentages of sociodemographic characteristics and perspectives and suggestions for DUFB marketing strategies.

Respondents were categorized based on whether they resided in metropolitan (metro) or nonmetropolitan (nonmetro) counties according to the USDA’s rural classification [34]. Nonmetro counties include some combination of open countryside, rural towns (places with fewer than 5,000 people and 2,000 housing units), and urban areas with populations ranging up to 50,000 people that are not part of larger labor market areas (metro areas). Metro counties in New Mexico include Bernalillo, Doña Ana, San Juan, Sandoval, Santa Fe, Torrance, and Valencia counties. Perspectives of marketing tactics and tools and barriers to participating in DUFB were compared between survey respondents residing in metro and nonmetro counties using chi-square tests. The significance of comparisons between groups was set at *P* < 0.05.

## Results

The final sample included 1061 respondents. Demographic characteristics of survey respondents are shown in Table 3. Most were female (84%); 35–49 years of age (46%); and Hispanic, any race (51%) or non-Hispanic White (28%). More than 40% had completed some college or were college graduates. Nearly half (48%) reported an annual household income of less than $20,000 and more than 80% had an annual household income of less than $50,000. Survey respondents were from all 33 counties in New Mexico; 64% were from metro counties and 36% were from nonmetro counties.

**Table 3:** Demographic characteristics of survey respondents aged 18 years or older living in new Mexico and participating in the supplemental nutrition assistance program (SNAP) (*n* = 1061)

Characteristics	*n* (%)	Characteristics	*n* (%)
**Gender ** ^a^	884 (83%)	**Highest Education** ^**a**^	892 (84%)
Male	125 (14%)	Less than high school	93 (10%)
Female	743 (84%)	High school graduate or GED	293 (33%)
Other	5 (1%)	Some college or college graduate	390 (44%)
Prefer not to say	11 (1%)	More than college (some post-graduate, graduate, or professional degree)	96 (11%)
**Age** ^a^	892 (84%)	Prefer not to answer	20 (2%)
18–34 years	281 (32%)	**Annual Household Income** ^**a**^	887 (84%)
35–49 years	413 (46%)	Less than $20,000	428 (48%)
50–64 years	142 (16%)	$20,000 - $49,999	310 (35%)
> 64 years	56 (6%)	$50,000 or more	73 (8%)
**Race and Ethnicity** ^a^	884 (83%)	Prefer not to answer	76 (9%)
Native American, not Hispanic	102 (12%)	**Adults in the Household** ^**a**^	878 (83%)
Asian, not Hispanic	9 (1%)	1	322 (36%)
African American, not Hispanic	18 (2%)	2	351 (40%)
Hispanic, any race	450 (51%)	3	124 (14%)
		> 3	81 (10%)
White, not Hispanic	245 (28%)	**Children in the Household** ^**a**^	878 (83%)
Multiple races, not Hispanic	13 (1%)	0	219 (25%)
Race and ethnicity unknown	47 (5%)	1	201 (23%)
**Rural Classification** ^a^	876 (83%)	2	213 (24%)
Metro	564 (64%)	3	128 (14%)
Nonmetro	312 (36%)	> 3	117 (14%)

^a^ Frequency and percentage of the demographic characteristic category (i.e., Gender, Age, Race and Ethnicity, etc.) reflect the number and percentage of survey respondents who answered each optional demographic characteristic question out of the total number of survey respondents (*n* = 1061)

Table 4 shows characteristics of survey respondents. Among all respondents, 943 (89%) answered the survey in English, and 118 (11%) answered the survey in Spanish. Most (77%) had participated in SNAP for more than 12 months. 65% (*n* = 689) reported being aware of the DUFB program, however, only 40% (*n* = 421) had ever participated in DUFB. Three fourths (75%) indicated that they participated in or received benefits from other assistance programs, including school lunch or breakfast programs (44%), Special Supplemental Nutrition Program for Women, Infants, and Children (WIC) or the WIC Farmers’ Market Nutrition Program (34%), Summer Meals Programs (28%), and/or Temporary Assistance for Needy Families (16%). Among survey respondents that had participated in DUFB, 41% had participated for more than 12 months.

**Table 4:** Characteristics of survey respondents aged 18 years or older living in new Mexico and participating in the supplemental nutrition assistance program (SNAP) (*n* = 1061)

Characteristics	*n*^a^ (%)	Characteristics	*n*^a^ (%)
**Survey Language**	1061 (100%)	**Other program participation** ^b^	798 (75%)
English	943 (89%)	WIC or WIC Farmers’ Market Nutrition Program	267 (34%)
Spanish	118 (11%)	TANF (Temporary Assistance for Needy Families)	128 (16%)
**Aware of DUFB**	1061 (100%)	FDPIR (Food Distribution Program on Indian Reservations)	34 (4%)
Yes	689 (65%)	Medicaid or CHIP	539 (7%)
No	372 (35%)	Head Start Program	56 (7%)
**Ever Participated in DUFB**	1061 (100%)	Seniors Farmers’ Market Nutrition Program	64 (8%)
Yes	421 (40%)	Summer Meals Programs (Summer Foodservice Program, Child and Adult Care Food Program)	227 (28%)
No	640 (60%)	School Lunch or Breakfast Program	349 (44%)
**Length of time participating in DUFB**	384 (36%)	Other	34 (4%)
< 1 month	69 (18%)	None of above	6 (1%)
1–5 months	84 (22%)		
6–12 months	74 (19%)		
> 12 months	157 (41%)		
**Length of time receiving SNAP**	534 (50%)		
< 1 month	32 (6%)		
1–5 months	43 (8%)		
6–12 months	48 (9%)		
> 12 months	411 (77%)		

^a^ Number of responses varied by question due to missing data (incomplete responses or skipping questions)

^b^Participants could select more than one option

**Fig. 2:**
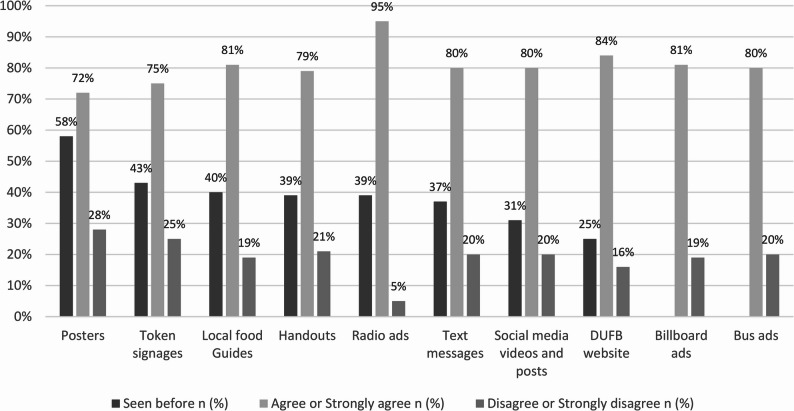
Awareness of DUFB marketing tactics and tools and survey respondents’ agreement (%) for each tactic and tool regarding encouraging their participation in DUFB.^a^ ^a^Bus advertisement was only implemented in Albuquerque, NM and is no longer available; billboards were implemented just three days before the research was conducted. The percent of survey respondents who had seen bus and billboard advertisements before was therefore not evaluated in the survey

Awareness of DUFB marketing tactics and tools and agreement regarding encouraging DUFB participation are shown in Fig. 2, listed in descending order of awareness percentage. Overall, the awareness of DUFB marketing tactics and tools was low. Tools with the highest awareness were posters (58%), token signage (43%), and the local food guide (40%), but at least 72% of respondents agreed or strongly agreed that each marketing tactic or tool would encourage them to participate in DUFB (Fig. 2). The highest-ranking tools were radio advertisements (95%), the DUFB NM website (84%), billboard advertisements (81%), and text messages (81%).

### Posters

A total of 1,012 participants indicated wanting to see posters at additional locations such as participating DUFB markets (77%); public aid offices, WIC clinics, and cooperative extension offices (58%); food banks, food pantries, and soup kitchens (58%); community centers (53%); and schools and childcare centers (53%).

### Local food guide

Respondents suggested distributing more copies of the Local Food Guide (*n* = 29, 3%) and adding a list of participating locations to the Local Food Guide (*n* = 24, 2%).

### Radio ads

Most respondents mentioned that doubling up or getting twice as much stood out in the radio ad (*n* = 213, 20%) followed by food costs and saving money (*n* = 102, 10%).

### Text messages

Among 925 respondents, 82% preferred receiving text messages in English, and 12% preferred receiving text messages in Spanish. In addition, 570 participants (97% of the respondents who had not received text message before) provided their phone numbers to receive GoodFoodNM texts while completing the survey, indicating strong interest in the program and willingness to engage through mobile communication.

### Social media videos and posts

Among 927 respondents, SNAP participants suggested that they would like to see social media posts and videos on Facebook (80%), TikTok (46%), Instagram (46%), YouTube (45%), and Snapchat (25%). Respondents also shared effective strategies such as sharing and tagging (*n* = 37, 4%) and using stories and real people in content (*n* = 25, 3%). Survey respondents’ preferred content type was videos (*n* = 36, 4%), pictures (*n* = 8, 1%), and links (*n* = 4, 0%).

### Metro/nonmetro differences

Differences in awareness and perspectives of tactics and tools were examined between survey respondents residing in metro and nonmetro counties (Table 5). Overall, there were statistically significant differences in awareness of radio advertisement, token signage, handouts, local food guide, videos and social media posts, and the DUFB website between respondents from metro and nonmetro counties (*P* < 0.05). Specifically, respondents in nonmetro counties reported higher-than-expected awareness of those marketing tools, indicating they were significantly more likely to have seen marketing tools and tactics before compared to their metro counterparts. Respondents in nonmetro counties were also significantly more likely to agree or strongly agree that posters, token signage, social media posts, bus advertisements, and the DUFB website would encourage them to participate in the DUFB program than those from metro counties (*P* < 0.05).

**Table 5:** Awareness and survey respondent agreement (%) for each tactic and tool regarding encouraging their participation in DUFB between survey respondents from metro (*n* = 564) and nonmetro (*n* = 312) counties

Marketing tools and tactics	Seen before *n* (%)^a^		X^2^*P*-Value	Agree or Strongly Agree *n* (%)^a^		Disagree or Strongly Disagree *n* (%)^a^		X^2^*P*-Value
	Metro	Nonmetro		Metro	Nonmetro	Metro	Nonmetro	
Posters	355 (63%)	187 (60%)	0.38	395 (70%)	240 (77%)	169 (30%)	72 (23%)	0.03^*^
Token signage	231 (41%)	156 (50%)	0.01^*^	418 (74%)	252 (81%)	146 (26%)	60 (19%)	0.02^*^
Local food Guide	209 (37%)	144 (46%)	0.009^*^	451 (80%)	263 (84%)	113 (20%)	49 (16%)	0.14
Handouts	203 (36%)	147 (47%)	0.001^*^	442 (78%)	256 (82%)	122 (22%)	56 (18%)	0.16
Radio ads	203 (36%)	144 (46%)	0.003^*^	548 (97%)	293 (94%)	16 (3%)	19 (6%)	0.08
Text messages	203 (36%)	128 (41%)	0.14	453 (80%)	256 (82%)	111 (20%)	56 (18%)	0.45
Social media videos and posts	152 (27%)	125 (40%)	< 0.0001^*^	442 (78%)	267 (86%)	122 (22%)	45 (14%)	0.005^*^
DUFB website	107 (19%)	115 (37%)	< 0.0001^*^	462 (82%)	276 (88%)	102 (18%)	36 (12%)	0.02^*^
Billboard ads^b^	/	/	/	452 (80%)	264 (85%)	112 (20%)	48 (15%)	0.07
Bus ads^b^	/	/	/	439 (78%)	263 (84%)	125 (22%)	49 (16%)	0.03^*^

^a^ The question “Which county in New Mexico does the participant live?” was optional in the survey, which resulted in missing data. The total number of all participants who answered the awareness and agreement questions was greater than those who also answered about the county they lived in

^b^ Bus advertisement was only implemented in Albuquerque, NM and is no longer available; billboards were implemented just three days before the research was conducted. The percent of survey respondents who had seen bus and billboard advertisements before was therefore not evaluated in the survey

^*^statistical significance is indicated when *P* < 0.05

### Barriers to participating DUFB

Shown in Table 6, the most common barriers reported by SNAP participants who had never used DUFB (*n* = 538) were not knowing about DUFB (57%), not knowing how to use DUFB (36%), and/or no DUFB location nearby (21%). There were no significant differences in barriers in participating in the DUFB program between metro and nonmetro respondents (all *P*-values were > 0.1).

**Table 6:** Barriers to participating in DUFB among SNAP participants that have never used DUFB (*n* = 538) and differences between those from metro and nonmetro counties

	All (*n* = 538^a^)	Metro (*n* = 328^a^)	Nonmetro (*n* = 178^a^)	X^2^*P*-Value
Did not know about DUFB	310 (57%)	187 (57%)	109 (61%)	0.36
Do not know how to use DUFB	194 (36%)	125 (38%)	61 (34%)	0.39
No DUFB locations nearby	114 (21%)	62 (19%)	43 (24%)	0.17
Cannot get to DUFB locations nearby	79 (15%)	49 (15%)	27 (15%)	0.95
Not interested in purchasing fruits and vegetables	15 (3%)	12 (4%)	3 (2%)	0.21

^a^ The question “Which county in New Mexico does the participant live?” was optional in the survey, which resulted in missing data. The total number of all participants who answered this barrier question was greater than those who also answered about the county they lived in

## Discussion

This study sought to understand New Mexico SNAP participants’ awareness and perceptions of DUFB marketing tactics and tools, including differences by metro and nonmetro location, and barriers to participating in DUFB. Overall, current DUFB marketing tactics and tools had relatively low awareness, with less than half of survey respondents reporting having previously seen most of the tools. Despite the lack of awareness of most tactics and tools, at least 72% of respondents agreed that all marketing tactics and tools would encourage them to participate in DUFB. Radio advertisements, the DUFB website, billboards, and text messages ranked as the most effective tools, with agreement ratings ranging from 81% to 95%.

SNAP participants in nonmetro counties reported significantly higher awareness of radio advertisements, token signage, handouts, the local food guide, social media videos and posts, and the DUFB website compared to those in metro counties. Therefore, potential disparities may exist in the distribution of tactics and tools or availability of tactics and tools in metro versus nonmetro communities. Differences could also be explained by higher use of radio in nonmetro areas, and/or that marketing approaches in nonmetro areas may have been more effective [35]. In addition, nonmetro respondents were significantly more likely than metro respondents to agree that marketing tools like posters, token signage, social media videos and posts, bus advertisements, and the DUFB website would encourage their participation in the program. While DUFB marketing tactics and tools were considered successful in promoting DUFB participation, results still show significant barriers to DUFB participation, particularly lack of awareness and understanding of the program. This finding is consistent with previous studies on DUFB and other food incentive programs[6–8, 36–39]. Thus, it is important to increase program awareness through effective marketing strategies and provide clear and accessible information on how to use DUFB in marketing tactics and tools.

Posters are effective marketing tools and, in this study, they were the only marketing tool evaluated that had been seen by more than half of respondents [40]. One potential reason for the high awareness rate is that some participants were recruited directly from farmers’ markets where posters promoting DUFB were more likely to be prominently displayed. Token signage, the local food guide, and handouts also showed comparatively higher awareness, likely because SNAP participants at participating DUFB locations can access this information on the spot and immediately use DUFB.

The relatively low awareness of social media posts and the DUFB website suggests these digital tools may not be effectively reaching the target audience. Potential barriers to receiving online marketing advertisements or information may include limited exposure, ineffective targeting, or low engagement with current outreach efforts [7, 41]. Respondents indicated a preference for seeing DUFB advertisements on platforms such as Facebook, TikTok, Instagram, and YouTube, consistent with previous studies [39, 42]. An advantage of social media marketing is that users share their experience via posts and tagging with a wide network, extending the reach of information about the program.

Differences in awareness and perceived effectiveness of DUFB marketing tools and tactics were found between survey participants residing in metro and nonmetro areas of New Mexico. Nonmetro respondents reported greater awareness of six marketing tools and tactics (radio advertisement, token signage, handouts, local food guide, social media videos and posts, DUFB website) compared to their metro counterparts. Thus, those marketing tactics and tools may be more effective in nonmetro counties because traditional marketing strategies align better with media habits of people in nonmetro counties [39]. 

Survey respondents strongly agreed that all DUFB marketing tactics and tools evaluated would encourage DUFB program participation and radio advertisements, website, billboard advertisements, and text messages were most favored. Radio advertisements are forms of passive engagement that are effective marketing tools in public health and nutrition interventions [43, 44]. Radio stations also have strong community ties, especially in nonmetro areas [45, 46]. Billboard advertisements are hard to miss and consistently convey messages to people during daily commutes, thus are useful to reach the general population [47, 48]. Tailored text messages serve as an immediate and direct communication tool, reaching individuals instantly and allowing them to take action with ease [49]. In the future, these marketing tactics and tools might be considered first in efforts to increase awareness and participation in food incentive programs like DUFB.

The strengths of the current study include the comprehensive evaluation of ten marketing strategies, tools and tactics currently used for the New Mexico DUFB program. In addition, the NMFMA and DUFB program leadership who have first-hand experience with the program and its target population assisted in developing the survey. We also assessed differences in perspectives of tactics and tools between SNAP participants residing in metro and nonmetro counties to inform targeted marketing and communication approaches.

Limitations of this study should also be noted. A cross-sectional survey was used to collect data which can only show the sample’s attitudes and behaviors at a single time point. Survey respondents were recruited through GoodFoodNM text messages, social media, and flyers posted at eight DUFB-participating farmers’ markets across the state which may have led to an overrepresentation of DUFB participants in the survey and limit generalizability of study findings. Further, most recruitment occurred through text messages and social media posts, which may have limited reach in some rural areas with less reliable internet or mobile access. Indeed, the sample also included predominantly female respondents (84%) and respondents from metro counties (64%). More non-female respondents and respondents from nonmetro counties should be recruited in future studies to generate more representative results.

Further, survey questions were not formally pretested or validated. All information was self-reported including New Mexico residency and SNAP participation status, which may affect the accuracy of the results and could introduce biases such as recall bias and social desirability bias. Additionally, the survey was lengthy, evaluating 10 marketing tactics and tools, and took approximately 15–20 min to complete. The median completion time was 11.5 min, though some respondents started the survey but did not complete it in one sitting. Since respondents could stop taking the survey at any time and several questions were not required, the number of responses for each question was different, resulting in missing data for some analyses. Finally, survey respondents were presented with examples of marketing tactics and tools and did not evaluate the full tactic or tool in some cases.

Last, we acknowledge online surveys can be affected by fraudulent and bot responses; however, the research team thoroughly reviewed survey data, used stringent Qualtrics criteria to exclude duplicates and bots, and reviewed open-ended survey question responses to further exclude duplicates and suspicious responses. Additionally, two attention-check questions were included and participants that did not accurately answer both questions were excluded.

## Conclusion

This study investigated SNAP participants’ perceptions of DUFB marketing tactics and tools used in New Mexico and found that radio advertisements, websites, billboard advertisements, and text messages were the most favorable tactics and tools to increase awareness and usage of the program. Based on differences found between metro and nonmetro survey respondents, and barriers reported in accessing DUFB, different marketing approaches may be needed to target potential participants by geographic location. These findings highlight the importance of selecting marketing strategies best suited for local contexts to enhance the reach and potential participation in food incentive programs like DUFB. Future research can build on this work by exploring strategies that promote long-term nutrition incentive program engagement and by developing culturally tailored and location-specific marketing strategies to more effectively reach diverse participant populations.

## Supplementary Information

Supplementary Material 1.

## Data Availability

The datasets generated and analyzed during the current study are not publicly available due to participants’ consent restricting the sharing of their information but de-identified, non-confidential datasets are available from the corresponding author on reasonable request.

## Abbreviations

DUFB: Double Up Food Bucks
SNAP: Supplemental Nutrition Assistance Program
NMFMA: New Mexico Farmers’ Marketing Association
Metro: Metropolitan
Nonmetro: Nonmetropolitan
WIC: Women, Infants, and Children
