# Supplementary material for: Marketing health by geographic location: improving awareness of the New Mexico Double Up Food Bucks program
Source: BMC Nutr. 2026 Feb 18;12:48. doi: 10.1186/s40795-026-01261-z (PMC12964727; doi:10.1186/s40795-026-01261-z)
Supplement: Supplementary file 1 — Supplementary Material 1. [file 40795_2026_1261_MOESM1_ESM.docx]

**Double Up Food Bucks Survey**

Eligibility questions:

1.Are you a New Mexico resident?

- Yes
- No (end of the survey)

2.Are you a current Supplemental Nutrition Assistance Program (SNAP) recipient?

- Yes
- No (end of the survey)

3.Are you at least 18 years of age?

- Yes
- No (end of the survey)

Screening questions:

4. Do you know about the Double Up Food Bucks program?

- Yes
- No

5. Have you ever participated in the Double Up Food Bucks program?

- Yes (skip 22- 26)
- No (skip 16-21)

The following questions will ask you about Double Up Food Bucks communication and marketing messages and strategies that have been used in New Mexico since 2016.

6. Radio ads: Add an example

Please listen to the recording.

1. Have you heard a Double Up radio ad like this before?

- Yes
- No

1. This radio ad would encourage you to participate in the Double Up Food Bucks.

- Strongly disagree
- Disagree
- Agree
- Strongly agree

1. What stands out to you about the radio ad?
2. What type of radio stations do you listen to?

7. Community posters:


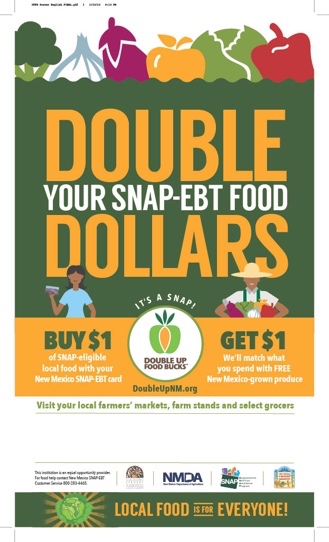

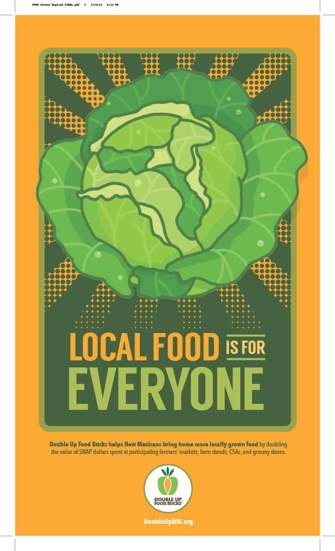

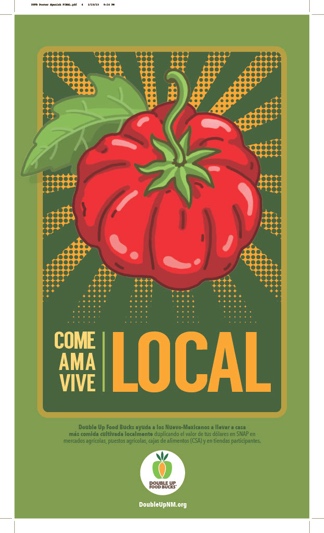


1. Have you seen any of these posters before?

- Yes
- No

1. These posters would encourage you to participate in Double Up Food Bucks.

- Strongly disagree
- Disagree
- Agree
- Strongly agree

1. What do you like about these posters?
2. What improvement could be made to these posters?
3. Where would you like to see these posters?

8. Double Up Food Buck program Token signage


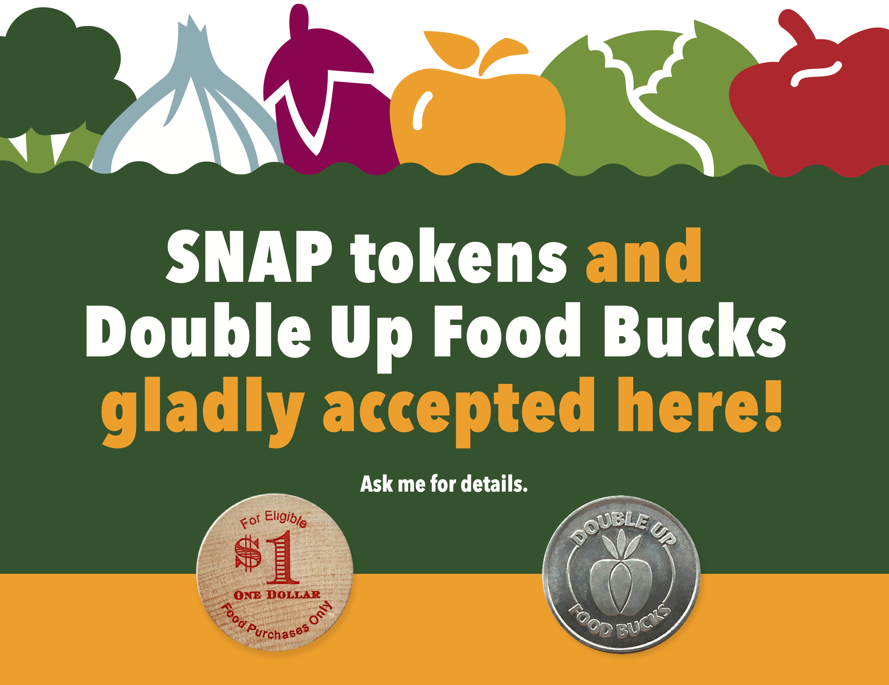


1. Have you seen this sign before?

- Yes
- No

1. This sign would encourage you to participate in Double Up Food Bucks.

- Strongly disagree
- Disagree
- Agree
- Strongly agree

9. Handouts:


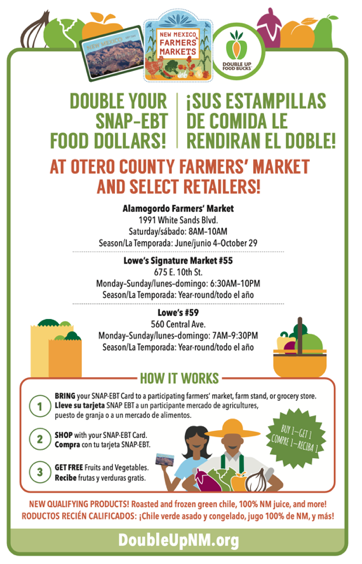

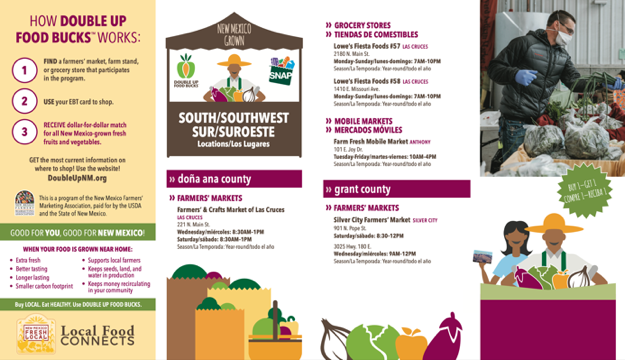


1. Have you seen any of these handouts before?

- Yes
- No

1. These handouts would encourage you to participate in Double Up Food Bucks.

- Strongly disagree
- Disagree
- Agree
- Strongly agree

1. Do you have any suggestions to make these handouts better?
2. Where would you like to receive these handouts?

10. Local food guide:


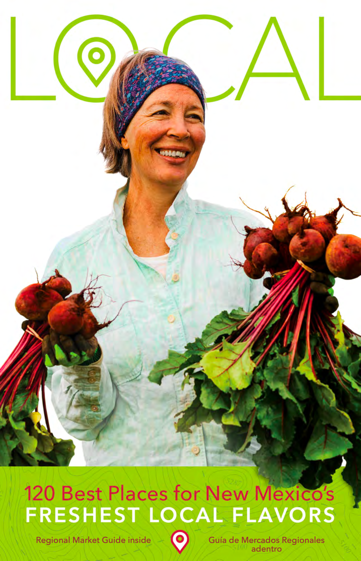

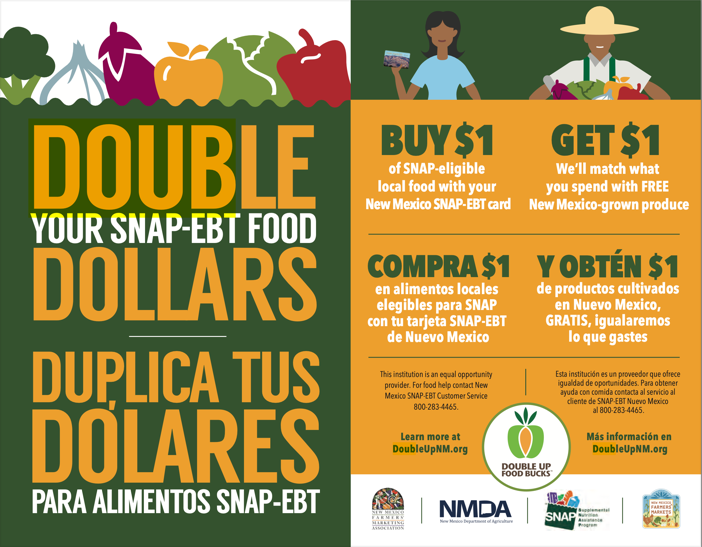


1. Have you seen this local food guide before?

- Yes
- No

1. This local food guide would encourage you to participate in Double Up Food.

- Strongly disagree
- Disagree
- Agree
- Strongly agree

1. Do you have any suggestions to make this local food guide better?
2. Where would you like to receive this local food guide?

11. Videos and posts on social media:


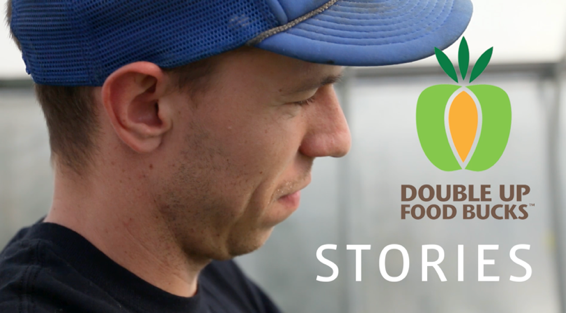

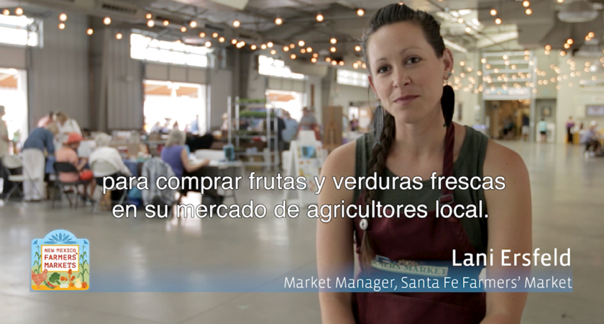

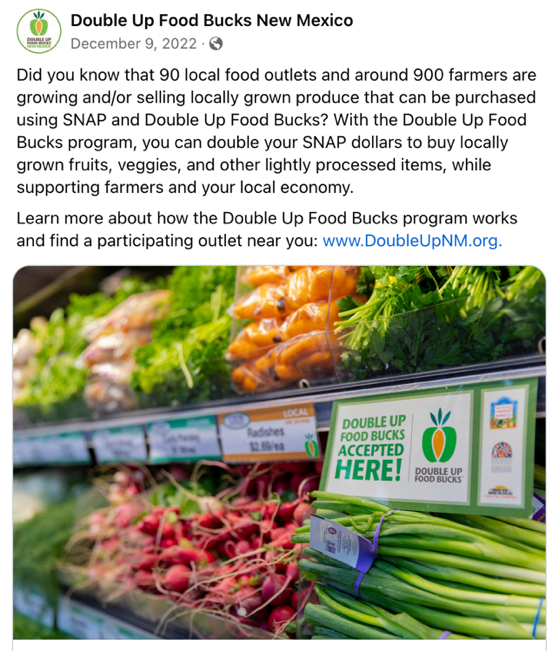

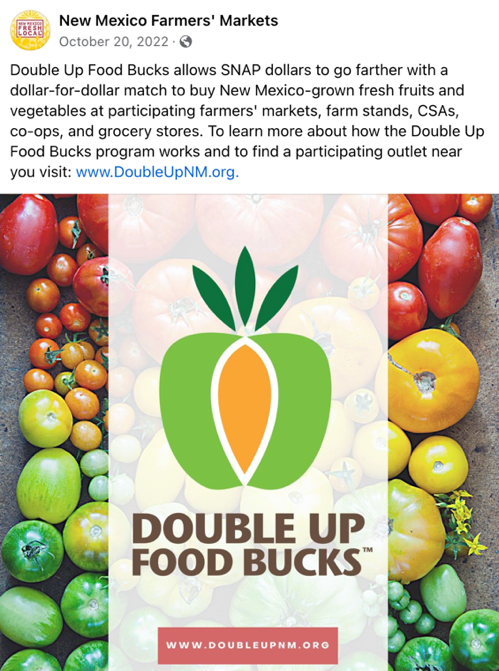


1. Have you seen any of these videos on social media before?

- Yes
- No

1. These videos and social media posts would encourage you to participate in Double Up Food Bucks.

- Strongly disagree
- Disagree
- Agree
- Strongly agree

1. What social media platforms would you like to see advertisements for the Double Up Food Bucks program in New Mexico? You can select more than one.

Facebook

Tik Tok

Instagram

YouTube

Reddit

X (formerly known as Twitter)

LinkedIn

Snapchat

Other:_______________________

1. How can social media be used to get more people to participate in Double Up Food Bucks?

12. Bus ads:


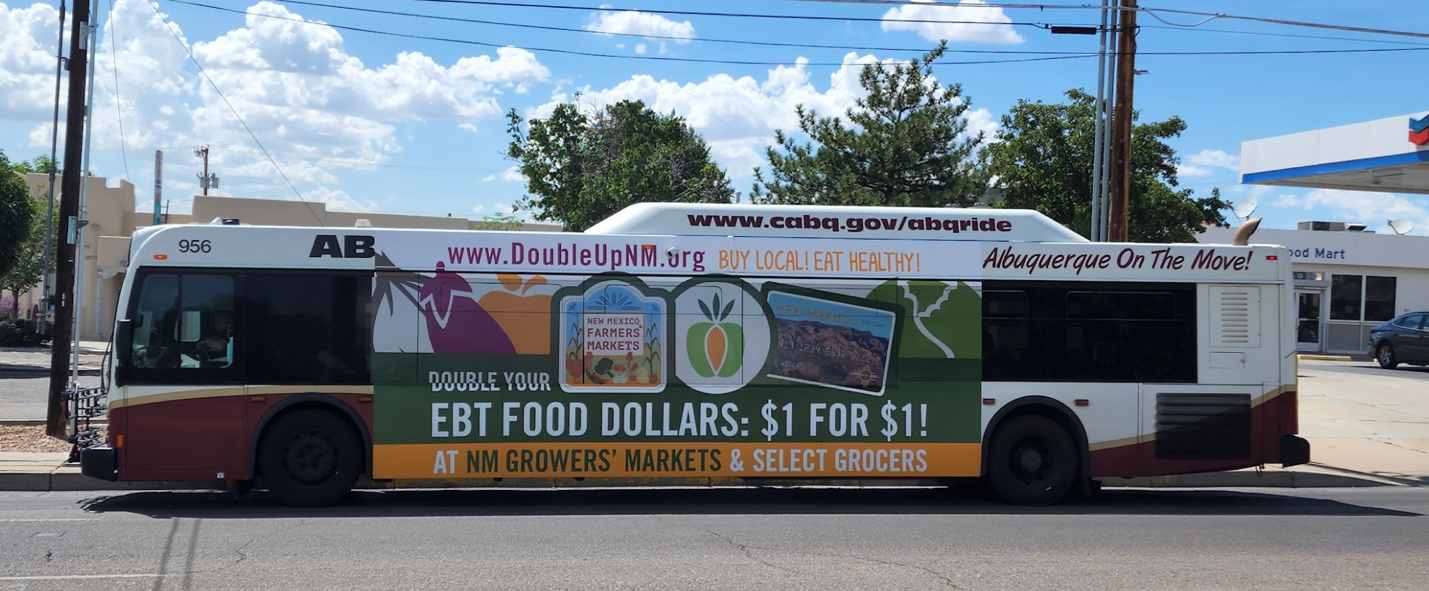


1. This bus ad would encourage you to participate in Double Up Food Bucks.

Strongly disagree

Disagree

Agree

Strongly agree

13. Billboard ads: add a picture here

1. This Billboard ad would encourage you to participate in Double Up Food Bucks.

- Strongly disagree
- Disagree
- Agree
- Strongly agree

14. Website:

(1) Have you ever visited the Double Up Food Bucks website? <https://doubleupnm.org/>

- Yes
- No

(2) The website would encourage you to participate in Double Up Food Bucks.

- Strongly disagree
- Disagree
- Agree
- Strongly agree

15. Free health texting messages:
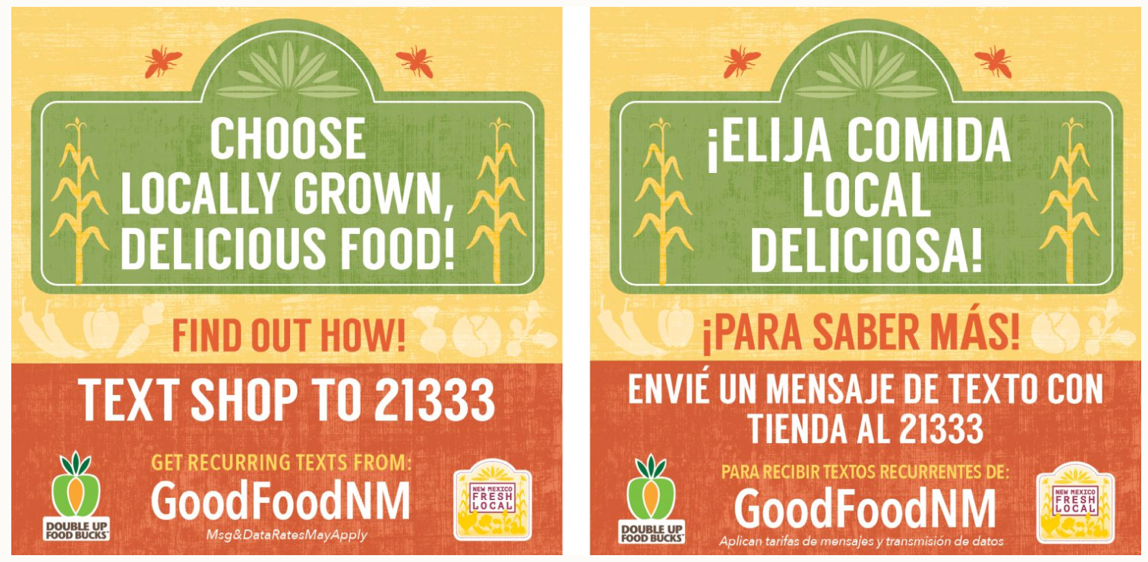


(1) Have you received Double Up Food Bucks text messages through “GoodFoodNM”?

- Yes
- No

(2) Text messages would encourage you to participate in Double Up Food Bucks.

- Strongly disagree
- Disagree
- Agree
- Strongly agree

16. Which language do you prefer to see or hear information about Double Up Food Bucks?

English

Spanish

No preference

Other: .

(Key questions for Double Up Food Bucks participants)

17. How did you first learn about the Double Up Food Bucks program?

Text messages

Local food guide

Radio ads

Bus ads

Handouts (Flyer, Handbills, and Brochures)

Double Up Food Bucks website

Social media posts and ads

Coupons

Farmers’ market signage (Flyers and Poster)

Friend or family

Food pantry

Healthcare provider

Community group

Nutrition educator

Departments of Social Services office/ Field offices

Local farmers/producers/market managers

Newspaper or news station like KOB, KRQE, etc

Other: ____________________________________________________

17. How long have you participated in the Double Up Food Bucks program?

Less than 1 month

1-5 months

6 months to 1 year

More than 1 year

18. How has participating in Double Up Food Bucks changed the following?

|  | increased | Same (no change) | decreased |
| --- | --- | --- | --- |
| The amount of fruits and vegetables you eat |  |  |  |
| Ability to afford fruits and vegetables |  |  |  |
| Trying new fruits and vegetables |  |  |  |
| The quality of your diet |  |  |  |

19. Please select how much you agree with this statement. I buy more fruits and vegetables only when I can use Double Up Food Bucks

- strongly disagree
- disagree
- agree
- strongly agree

20. Please select “Blue”.

Red

White

Blue

Green

21. On average, how much do you spend on fruits and vegetables for your household per week?

Less than $5

Between $5 and $19.99

Between $20 - $34.99

Between $35 - $49.99

$50 or more

(Key questions for participants who participate SNAP but not Double Up Food Bucks)

22. What are the best way to reach you about food incentive programs like Double Up Food Bucks? You can select more than one.

Text messages

Local food guide

Radio ads

Bus ads

Handouts (Flyer, Handbills, and Brochures)

Double Up Food Bucks website

Social media posts and ads

Coupons

Farmers’ market signage (Flyers and Poster)

Friend or family

Food pantry

Healthcare provider

Community group

Nutrition educator

Departments of Social Services office/ Field offices

Local farmers/producers/market managers

Newspaper or news station like KOB, KRQE, etc

Other: ____________________________________________________

23. Why don’t you use Double Up Food Bucks? Please select all that apply.

I did not know about Double Up Food Bucks

There are no Double Up Food Bucks locations nearby

I can’t get to the Double Up Food Bucks location nearby

I don’t know how to use Double Up Food Bucks

I am not interested in purchasing fruits and vegetables

Other: _______________

24. Please select “Red”.

Red

White

Blue

Green

25. How long have you received SNAP?

Less than 1 month

1-5 months

6 months to 1 year

More than 1 year

26. On average, how much do you spend on fruits and vegetables for your household per week?

Less than $5

Between $5 and $19.99

Between $20 - $34.99

Between $35 - $49.99

$50 or more

(Key questions for all)

27. Which of the following can you buy with Double Up Food Bucks? You can select more than one.

Fruits

Vegetables

Potatoes

Nuts

Beans and legumes

Plants that produce food (i.e., vegetable starts)

Cut herbs

Herb plants

You can buy all of these items with Double Up Food Bucks!

28. Have you or anyone in your household received any of the following in the past 12 months? Please select all that apply:

WIC or WIC Farmers’ Market Nutrition Program

TANF (Temporary Assistance for Needy Families)

FDPIR (Food Distribution Program on Indian Reservations)

Medicaid or CHIP

Head Start Program

Seniors Farmers Market Nutrition Program

School Lunch or Breakfast Program

Summer Meals Program (for example, Summer Foodservice Program, Child and Adult Care Food Program)

Other:______________________________

29. Which of the following would you be interested in receiving? Select all that apply.

Nutrition education (for example, learning ways to make the most of your purchases, cooking classes)

Medical support from a dietitian (for example, learning how to eat to help manage chronic conditions like diabetes or heart disease)

Grocery store tours

Farm tours

Food preservation information (for example, canning, freezing, drying foods, etc.)

Produce Prescription

Meal delivery

Other ______________________

30. Are you interested in enrolling in GoodFoodNM texting? GoodFoodNM includes free weekly text messages that promote good food and healthy eating across New Mexico. Message and data rates may apply.

- Yes (directed to enter phone number)
- No

31. Please enter your phone number to receive GoodFoodNM texts:

(demographic questions for all)

Continue answering questions to enter to win a $ 50 merchandise card!

32. What is your age?

18-34 years

35-49 years

50-64 years

65 years or older

33. What is the highest level of education you have completed?

Less than high school

High school graduate or GED

Some college or college graduate

More than college (some post-graduate, graduate, or professional degree)

Prefer not to answer

34. What is your yearly household income?

Less than $20,000

$20,000 - $49,999

$50,000 or more

Prefer not to answer

35. How many adults and/or children live in your household?

Adults (age 18 or older) - count yourself here

Children (under 18 years of age)

36. What is your gender?

- Woman
- Man
- Other (please specify): _______________
- Prefer not to answer

37. What is your race? You may select more than one.

American Indian/Alaska Native

Asian

Black/African American

Hispanic, Latino, or Spanish origin

Native Hawaiian/Other Pacific Islander

White

Other (please specify): __________________________________

Prefer not to answer

38. Which County in NM do you live? .

39. How can we let more New Mexicans know about the Double Up Food Bucks program?

Please fill out the contact information below if you would like to enter the drawing to win a $50 gift card. This will not allow research team to trace your answer.

| First Name |  |
| --- | --- |
| Email Address |  |

You have reached the end of the survey. When the survey ends, we will draw 5 participants to win a $50 merchandise card.

Double Up Food Bucks matches SNAP (food stamps) benefits on New Mexico-grown produce at participating outlets, making New Mexico-grown produce available at half off regular price for SNAP customers! Anyone receiving SNAP benefits is automatically eligible.

People interested in signing up for SNAP can find out more information by visiting <https://www.yes.state.nm.us> or calling 1-800-283-4465. More information can be found at <https://doubleupnm.org/>

Thank you for your participation!

40. Would you like to participate in a follow-up interview about Double Up Food Bucks marketing materials? This interview will randomly select 10 participants and each interviewee will receive a $40 merchandise card for participating.

- Yes (go to the recruitment of interview website)
- No
